# Knockdown of PHF5A Inhibits Migration and Invasion of HCC Cells via Downregulating NF-*κ*B Signaling

**DOI:** 10.1155/2019/1621854

**Published:** 2019-01-15

**Authors:** Qing Yang, Jianwen Zhang, Shilei Xu, Changchang Jia, Wei Meng, Hui Tang, Xiaomei Zhang, Yi Zhang, Binsheng Fu

**Affiliations:** ^1^Department of Hepatic Surgery and Liver transplantation Center of the Third Affiliated Hospital, Organ Transplantation Institute, Sun Yat-sen University and Organ Transplantation Research Center of Guangdong Province, Guangzhou 510630, China; ^2^Department of General Surgery, The Third Affiliated Hospital, Sun Yat-Sen University, Guangzhou 510530, China; ^3^Guangdong Key Laboratory of Liver Disease Research, The Third Affiliated Hospital of Sun Yat-sen University, Guangzhou 510630, China; ^4^Guangzhou Clinical Research and Translation Center for Liver Disease, The Third Affiliated Hospital of Sun Yat-sen University, Guangzhou 510630, China

## Abstract

**Background:**

Inflammation is the major risk factor for the progression of hepatocellular carcinoma (HCC), and the nuclear factor-*κ*B (NF-*κ*B) signaling plays the central role in the inflammation process. However, the activated mechanism of NF-*κ*B signaling in HCC is unclear.

**Methods:**

The expression of PHF5A is examined by qPCR, western blotting, and immunohistochemistry (IHC) assay. The potential of PHF5A (PHD-finger domain protein 5a) for migration and invasion is examined by wound healing and Transwell assay. Luciferase reporter assay, western blotting, and qPCR were applied to explore the mechanism by which PHF5A is involved in progression of HCC.

**Results:**

PHF5A was significantly upregulated in HCC tissues and cells. Downregulation of PHF5A inhibits the migration and invasion of HCC cells. Further study demonstrated that PHF5A is implicated in HCC progression through NF-*κ*B signaling. In addition, blocking the NF-*κ*B signaling can weaken the stimulatory effect of PHF5A on migration and invasion of HCC cells.

**Conclusion:**

PHF5A expression is upregulated in HCC tissues, and depletion of PHF5A inhibits the migration and invasion of HCC cells. Further experiments demonstrated that PHF5A is implicated in NF-*κ*B signaling and knockdown of PHF5A downregulates the activity of NF-*κ*B pathway to inhibit the tumor progression. The above results provide the evidence that PHF5A plays an indispensable role in progressive effect of NF-*κ*B pathway in HCC and may be a novel therapeutic target of HCC.

## 1. Introduction

Liver cancer is evaluated to be the sixth most commonly diagnosed cancer and the fourth leading cause of cancer death worldwide in 2018, with about 841,000 new cases and 782,000 deaths annually [1]. China alone is responsible for approximately 50% of the total number of new cases and deaths, and 70% to 90% of primary liver cancers are hepatocellular carcinoma (HCC) [2]. To HCC patients, the dominant cause of death is tumor progression induced by invasiveness and metastasis. Despite the fact that great efforts have been made to investigate the mechanism by which tumors obtain the potential of invasiveness and metastasis, the underlying mechanism of HCC metastasis remains unclear [3].

90% of HCC patients have undergone chronic liver inflammation, fibrosis, and/or subsequent cirrhosis [4, 5], and inflammation is the major risk factor for the progression of HCC. It has been established that the nuclear factor-*κ*B (NF-*κ*B) transcription factor family plays the central role in the inflammation process [6–8]. NF-*κ*B was first identified as B cell-specific transcription factor in 1986 [9, 10]. Subsequent studies demonstrated that NF-*κ*B signaling is constitutively active in both cancer cells and tumor microenvironment [11]. The NF-*κ*B signaling includes two general types: the classical/canonical and the alternative/noncanonical pathways. In the classical/canonical pathway, the inhibitor of NF-*κ*B (I*κ*B) protein, I*κ*B*α*, combines with NF-*κ*B complexes to sequester them in cytoplasm and inhibit them binding with DNA. Once a stimulus appears, the I*κ*B*α* is phosphorylated by the I*κ*B kinase (IKK) complex, IKK*β*, to elevate their ubiquitylation, and sequently proteasome-mediated degradated, which leads to NK-*κ*B (in classical pathway, they are p50-p65 complex) nuclear localization to activate the NK-*κ*B signaling and improve the transcription of downstream genes, including* slug* and* matrix metalloproteinase 9 (MMP9)* [12–14]. However, the activated mechanism of NF-*κ*B signaling in HCC is unclear.

PHD-finger domain protein 5a (PHF5A) is a highly conserved protein from yeast to human. PHF5A harbors 110 amino acids, a characteristic PHD-domain, and is ubiquitously expressed in the nucleus [15]. Previous studies suggested that PHF5A plays an indispensable role in embryo formation and tissue morphogenesis and regulates the transcriptional elongation of genes involved in cell pluripotency and differentiation [16, 17]. Subsequent studies showed that PHF5A is involved in alternative splicing and is essential for SF3b spliceosome stability and can link the spliceosome to histones [18]. Dysregulation of PHF5A is closely related to cancer progression [15, 19, 20]. However, the expression and function of PHF5A in HCC are unknown.

Herein, we found that PHF5A expression is upregulated in HCC tissues, and depletion of PHF5A inhibits the migration and invasion of HCC cells. Further experiments demonstrated that PHF5A is implicated in NF-*κ*B signaling and knockdown of PHF5A downregulates the activity of NF-*κ*B pathway to inhibit the migration and invasion of HCC cells. The inhibitor of NF-*κ*B pathway can effectively inhibit the stimulative effect of PHF5A on HCC cell migration and invasion. The above results suggest that PHF5A may serve as a prognostic factor and novel therapeutic target in HCC.

## 2. Materials and Methods

### 2.1. Cell Lines and Tissues

The human immortalized normal hepatocyte LO2 and HCC cell lines (MHCC97H, Hep3B, HepG2, Hub7, SNU-449, SNU-423, and BEL-7402) were cultured using Dulbecco's modified Eagle's medium (DMEM; Invitrogen, Carlsbad, CA, USA) added to 10% fatal bovine serum (FBS; HyClone, Logan, UT, USA) and 1% penicillin/streptomycin (HyClone, Logan, UT, USA). The human normal liver tissues and HCC tissues were collected from the Third Affiliated Hospital of Sun Yat-sen University between 2010 and 2013. Before collecting the specimens for research purpose, patient informed consent and approval from the Institutional Ethics Committee were both obtained.

### 2.2. Quantitative PCR (qPCR)

Total RNA was isolated using TRIzol reagent (Invitrogen, Carlsbad, CA, USA). cDNA was amplified using ABI 7500 Fast System (Applied Biosystems, Rockville, MD, USA). The gene of *α*-tubulin is used as the reference. The relative mRNA expression of genes was evaluated as the following formula: 2^-[(Ct  of  gene)-(Ct  of  *α*-tubulin)]^, and the Ct represents threshold cycle. The primers are as follows: PHF5A, forward, 5′-GCACTCTGGTGCGCATATGTGA-3′, reverse, 5′-GACAATCTTTGGGCAGCCATCTC-3′; MMP9, forward, 5′-GCCACTACTGT GCCTTTGAGTC-3′, reverse, 5′-CCCTCAGAGAATCGCCAGTACT-3′; Slug, forward, 5′-ATCTGCGGCAAGGCGTTTTCCA-3′, reverse, 5′-GAGCCCTCAG ATTTGACCTGTC-3′.

### 2.3. Plasmids

To overexpress PHF5A, full-length human PHF5A was amplified by PCR and then cloned into pMSCV plasmid. For knockdown of PHF5A, two human PHF5A-targeting shRNA sequences were cloned into the pSuper-retro-puro plasmid, respectively. The primers are as follows: sh#1: GCCTACTACTACCAGCAGAAA, and sh#2: TGTGTGATTTGTGACTCCTAT. The plasmids were transfected using Lipofectamine 3000 (Invitrogen, Carlsbad, CA, USA). The stable cell lines were screened using 0.5 *μ*g/mL puromycin for 10 days.

### 2.4. Western Blotting Assay

Western blotting was carried out in accordance with the methods described previously [21]. The primary antibodies were PHF5A, p65, p84, p-IKK-*β*, IKK-*β*, p-I*κ*B*α*, I*κ*B*α*, MMP9, and Slug (Abcam, Cambridge, MA, USA). All membranes were successively probed by HRP-conjugated secondary antibody (Abcam, Cambridge, MA, USA). And *α*-tubulin was served as the loading control.

### 2.5. Immunohistochemical (IHC) Assay

The slides were sectioned at 5 *μ*m, deparaffinized in xylene, and successively rehydrated in 100, 95, and 75% ethanol. Then the slides were immersed in Tris-EDTA for 10 min in a pressure boiler for antigen retrieval. Endogenous peroxidase activity was deleted using 3% hydrogen peroxide. Subsequently, the slides were blocked with 0.4% casein at room temperature and incubated using primary antibody overnight at 4°C. Finally, the slides were incubated with a biotinylated secondary antibody for 8 min and incubated with a streptavidin-HRP conjugate for 8 min at 37°C.

### 2.6. Wound Healing Assay

The cells were cultured in 6-well plates. When the confluence reached approximately 90%, the cell layer was scratched with 10 *μ*l pipette tip. 24 h later, the scratch was detected under microscope (CKX41; Olympus, Tokyo, Japan).

### 2.7. Transwell Matrix Penetration Assay

1 × 10^4^ cells were seeded into the upper chamber of the Transwell filter, which was coated with Matrigel (BD, Bedford, MA, USA) and added to DMEM (Invitrogen, Carlsbad, CA, USA), and the chamber was put into the 24-well plate that contained DMEM (Invitrogen, Carlsbad, CA, USA) and 10% FBS (HyClone, Logan, UT, USA). 24 hours later, the cells inside the upper layer of filter were removed using cotton swabs. The invaded cells adhering to the underlayer of the filter were fixed with 1% paraformaldehyde for 10 min and dyed with hematoxylin for 5 min and eventually examined under microscope (CKX41; Olympus, Tokyo, Japan) and the number of invaded cells was counted.

### 2.8. Transfection and Luciferase Reporter Assay

The reporter plasmid was constructed using the pGL3-Enhancer plasmid (Promega, Madison, WI, USA). Cells were cotransfected with plasmids expressing the firefly luciferase gene containing the NF-*κ*B response element and the Renilla luciferase gene at a 10:1 ratio. Transfection was carried out using Lipofectamine 3000 (Invitrogen, Carlsbad, CA, USA) according to the manufacturer's instructions. 48 hours after transfection, cell lysates were used for detecting firefly luciferase and Renilla luciferase activities using the dual luciferase reporter gene assay kit (Promega, Madison, WI, USA).

### 2.9. The Public Dataset Analysis

The genomic data containing the expression of PHF5A were downloaded from the publicly available dataset, the Cancer Genome Atlas (TCGA; https://cancergenome.nih.gov/). The microarray data containing the expression of PHF5A were downloaded from the publicly available dataset, the Gene Expression Omnibus (GEO; http://www.ncbi.nlm.nih.gov/geo).

### 2.10. Statistical Analysis

Every experiment was performed at least 3 times. Statistical analysis was carried out with SPSS 20.0 statistical software package, and the statistical significance between groups was analyzed using Student's* t*-test. Survival curves were plotted by the Kaplan-Meier and significantly analyzed using the log-rank test. The results are presented as mean ± standard deviation. P < 0.05 was considered as statistical significance.

## 3. Results

### 3.1. PHF5A Is Overexpressed and Closely Associated with Patient Survival in HCC

**Figure 1:**
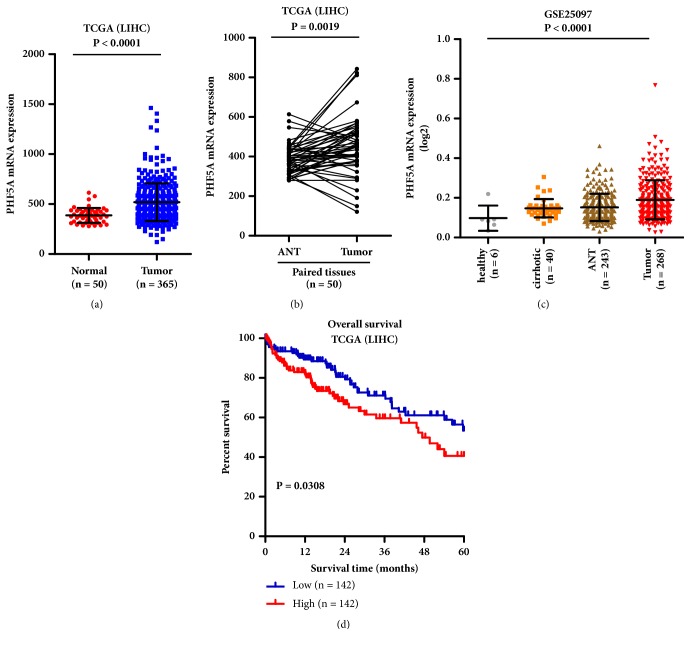
*PHF5A expression is upregulated in HCC by analyzing publicly available dataset.* (a) Relative expression of PHF5A in normal liver tissues is significantly lower than that in HCC tissues by analyzing the publicly available dataset TCGA. (b) PHF5A expression in 50 paired tissues by analyzing the publicly available dataset TCGA. (c) The PHF5A expression in the published HCC dataset NCBI/GEOGSE25097. (d) The overall survival for patients with high PHF5A is significantly shorter than that of patients with low PHF5A expression by analyzing TCGA dataset. LIHC: liver hepatocellular carcinoma.

To investigate the expression and role of PHF5A on the progression of HCC, firstly, we analyzed the expression of PHF5A in the publicly available dataset, the Cancer Genome Atlas (TCGA). The analysis demonstrated that the PHF5A expression is significantly upregulated in 365 primary HCC tissues compared to 50 normal liver tissues (Figure 1(a), P < 0.0001). We also analyzed the PHF5A expression in paired tissues. As shown in Figure 1(b), PHF5A is drastically upregulated in HCC tissues compared to matched adjacent normal tissues (ANT). Additionally, we further verify PHF5A expression in the published mRNA expression profile (GSE25097) from NCBI GEO (https://www.ncbi.nlm.nih.gov/geo/). Consistent with the above results, the PHF5A expression is increased according to the following order: healthy live, cirrhotic, ANT, and HCC tissues (Figure 1(c)). Next, Kaplan-Meier survival analysis demonstrated that the overall survival of HCC patients with high expression of PHF5A is significantly shorter than that of patients with low expression of PHF5A using the data from TCGA (Figure 1(d)).

**Figure 2:**
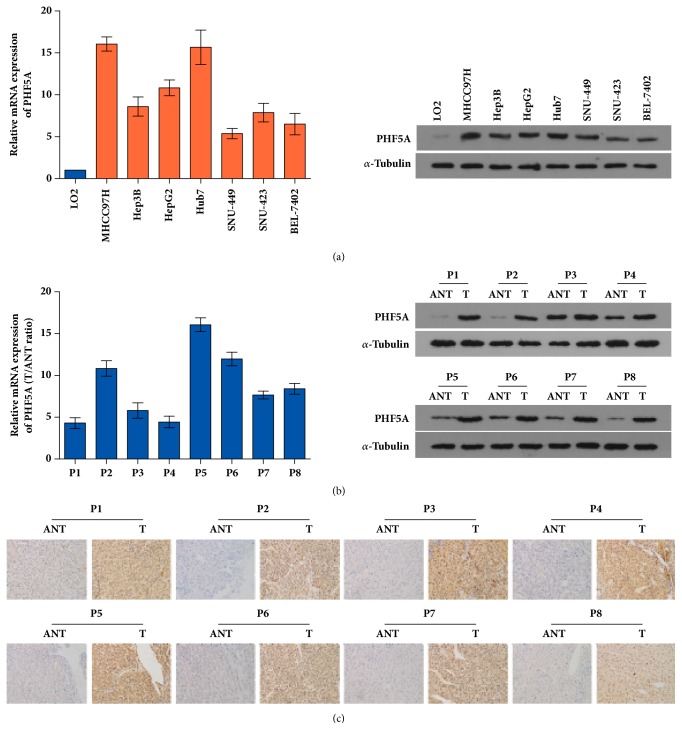
*PHF5A expression is drastically upregulated in HCC cell lines and fresh HCC tissues.* (a) PHF5A expression is significantly increased in HCC cell lines compared with immortalized normal liver cell LO2 by qPCR (left panel) and western blotting (right panel) assay. (b) qPCR (left panel) and western blotting (right panel) assay showed that PHF5A expression is dramatically upregulated in fresh HCC tissues compared with ANT. (c) IHC assay demonstrated that PHF5A is markedly increased in HCC tissues compared with ANT using paired tissue slides. ANT: corresponding adjacent normal tissues.

Subsequently, qPCR and western blotting assay showed that the expression of PHF5A is markedly increased in HCC cell lines (MHCC97H, Hep3B, HepG2, Hub7, SNU-449, SNU-423, and BEL-7402) compared to normal human immortalized hepatocyte LO2 cells on both mRNA and protein level (Figure 2(a)). Likewise, the expression of PHF5A is dramatically upregulated in fresh HCC tissues (T), while it is hardly detectable in the corresponding adjacent normal tissues (ANT) using qPCR, western blotting, and IHC assay (Figures 2(b) and 2(c)).

Altogether, the PHF5A expression is significantly increased in HCC tissues and cell lines, and the high level of PHF5A is closely correlated with poor survival of HCC patients.

### 3.2. Knockdown of PHF5A Inhibits Migration and Invasion of HCC Cells

**Figure 3:**
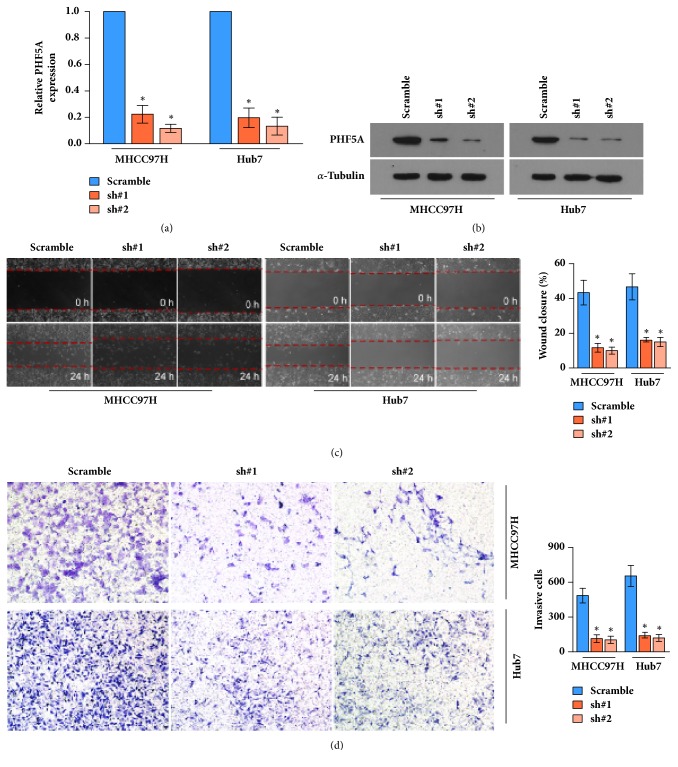
*Knockdown of PHF5A inhibits migration and invasion of HCC cells.* (a) qPCR assay of PHF5A in indicated stable cell lines. (b) Western blotting of PHF5A in indicated stable cell lines, and *α*-tubulin is served as the loading control. (c) Representative micrographs (left panel) and quantification (right panel) of wound healing assay both illustrated that inhibition of PHF5A downregulates migration of HCC cells. (d) Representative micrographs (left panel) and quantification (right panel) of Transwell matrix penetration assay suggest that knockdown of PHF5A can inhibit invasion of HCC cells. *∗* P < 0.05.

To explore the role of PHF5A in HCC cell migration and invasion, stably silencing PHF5A cell lines were constructed using MHCC97H and Hub7, in which PHF5A expression is higher than in that other cells (Figures 3(a) and 3(b)). Subsequently, the wound healing assay and Transwell assay were carried out. The results demonstrated that silencing of PHF5A inhibits the potential for migration and invasion of HCC cells (Figures 3(c) and 3(d)).

Collectively, our results suggest that PHF5A plays an important role in progression of HCC, and silencing of PHF5A inhibits migration and invasion of HCC cells.

### 3.3. PHF5A Is Involved in the NF-*κ*B Signaling Pathway

**Figure 4:**
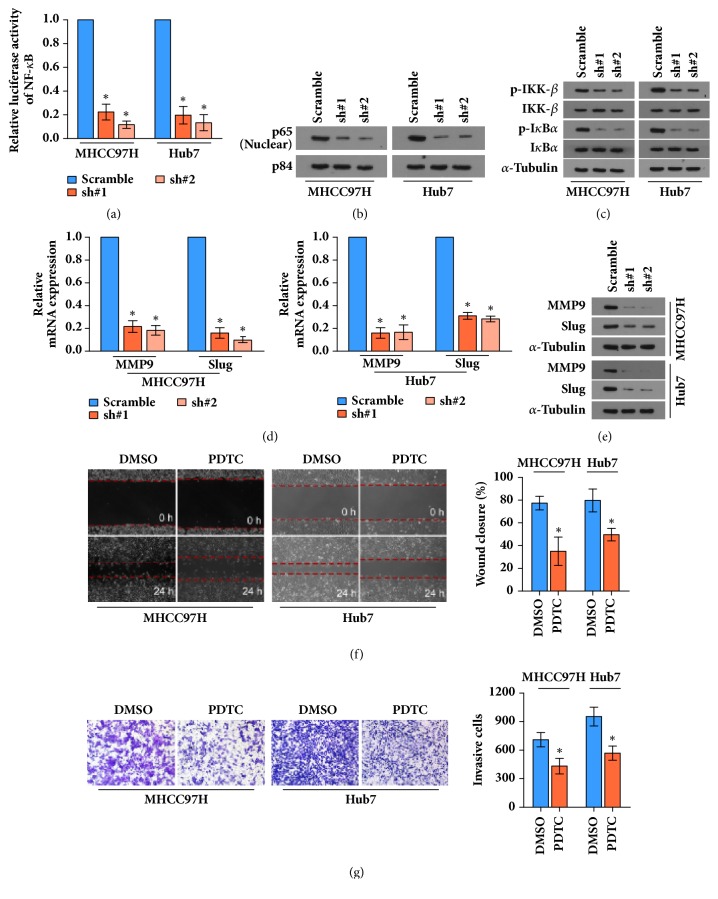
*PHF5A is involved in HCC progression via the NF-κB signaling.* (a) The luciferase reporter assay showed that transactivity of NF-*κ*B signaling is significantly inhibited in PHF5A-silenced cells. (b) The expression of p65 in cell nucleus is significantly decreased in PHF5A-silenced cells by western blotting, and p84 serves as the nucleus loading control. (c) The expressions of p-IKK-*β*, IKK-*β*, p-I*κ*B*α*, and I*κ*B*α* in total lysate of cells by western blotting assay, and *α*-tubulin serves as the loading control. (d) Relative mRNA expression of MMP9 and Slug is significantly decreased in PHF5A-silenced HCC cells by qPCR assay. (e) The expression of MMP9 and Slug is dramatically decreased in PHF5A-silenced HCC cells by western blotting assay. (f) Wound healing assay showed that blocking NF-*κ*B pathway can inhibit the stimulative effect of PHF5A on migration of HCC cells using PDTC. (g) Transwell matrix penetration assay showed that blocking NF-*κ*B pathway can inhibit the stimulative effect of PHF5A on invasion of HCC cells using PDTC. *∗* P < 0.05.

Since inflammation is essential for the progression of HCC and NF-*κ*B signaling plays the main role in the inflammation process, we further investigate whether PHF5A is involved in the NK-*κ*B pathway. As illustrated in Figure 4(a), the relative transactivity of NF-*κ*B signaling is significantly decreased in the PHF5A-silenced HCC cells. Besides, the level of p65 is significantly reduced in PHF5A-silenced cells (Figure 4(b)). Because the location of p65 is mainly influenced by the phosphorylation levels of IKK-*β* and I*κ*B*α*, we also observed that knockdown of PHF5A significantly inhibits the phosphorylation of IKK-*β* and I*κ*B*α* using western blotting assay (Figure 4(c)), suggesting that downregulation of PHF5A can inhibit the NF-*κ*B signaling. Furthermore, we examined the mRNA expression of NF-*κ*B downstream genes,* mmp9* and* slug*. The qPCR and western blotting assay both demonstrated that downregulation of PHF5A can inhibit their expression (Figures 4(d) and 4(e)). Eventually, we blocked the NF-*κ*B signaling in PHF5A-overexpressing cells by 10 *μ*M ammonium pyrrolidinedithiocarbamate (PDTC; Selleck, Houston, Texas, USA), which is a small molecular inhibitor of NF-*κ*B signaling by suppressing I*κ*B phosphorylation. The wound healing and Transwell assay showed that blocking NF-*κ*B signaling can significantly weaken the stimulatory effect of PHF5A on migration and invasion of HCC cells, respectively (Figures 4(f) and 4(g)), suggesting that NF-*κ*B signaling is essential for the stimulatory effect of PHF5A on HCC progression.

Collectively, downregulation of PHF5A can significantly inhibit the transactivity of NF-*κ*B signaling in HCC cells.

### 3.4. PHF5A Level Correlated with NF-*κ*B Signaling Activation in HCC

**Figure 5:**
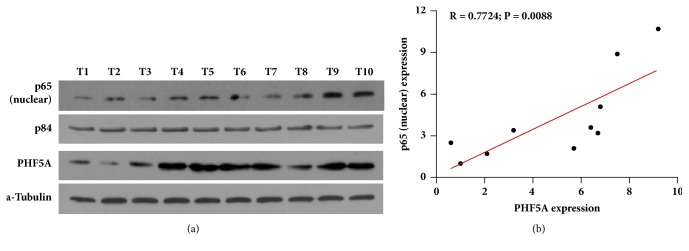
*PHF5A level is associated with activation of NF-κB signaling in clinical HCC tissues. *(a) The western blotting assay of p65 levels in nucleus and PHF5A expression in total cells. (b) The correlation analysis showed that p65 levels in nucleus are positively correlated with the PHF5A expression in total cells.

To evaluate whether PHF5A level is associated with activation of NF-*κ*B signaling in clinical HCC tissues, western blotting assay was applied to examine the PHF5A expression in total cells and p65 expression in cell nucleus. As shown in Figure 5, there is significantly positive correlation between PHF5A expression in total cells and p65 expression in nucleus. These results further support the hypothesis that PHF5A contributes to migration and invasion of HCC cells via activating the NF-*κ*B signaling.

## 4. Discussion

PHF5A is an important component of spliceosome [18], which suggests that PHF5A is involved in transcription regulation of different genes and dysregulation of PHF5A may induce the disorder of human body. Nimmakayala et al. showed that cigarette smoke extract can increase the expression of PHF5A and activates the pluripotency of pancreatic cells [22]. Zheng and his colleges demonstrated that upregulation of PHF5A leads to poor survival of breast cancer via inhibiting Fas-mediated apoptosis [20]. PHF5A is highly upregulated in lung adenocarcinoma and PHF5A knockdown can result in reducing cell proliferation and cell cycle arrest and contributes to cell apoptosis [18]. PHF5A facilitates recognition of exons with unusual C-rich 3' splice sites in human brain tumor and is required for cell viability [23]. In our study, we uncovered that PHF5A is upregulated in HCC cell lines and tissues, and knockdown of PHF5A can significantly inhibit the migration and invasion of HCC cells. Therefore, our study showed that knockdown of PHF5A may be an effective way to treat HCC. But this needs more evidence.

The NF-*κ*B signaling participates in many steps of cancer initiation and progression, such as cancer cell proliferation and survival, invasion, angiogenesis, and metastasis [12, 24–26]. He et al. showed that NF-*κ*B promotes HCC metastasis via enhancing epithelial-mesenchymal transition [27]. Tey et al. demonstrated that NF-*κ*B signaling is activated by nuclear MET to promote HCC tumorigenesis and metastasis [28]. We also found that the NF-*κ*B is activated in HCC and PHF5A plays an important role in the activation of NF-*κ*B signaling. Moreover, the inhibitor of NF-*κ*B can effectively inhibit the progression of HCC, suggesting that inhibition of NF-*κ*B signaling may be an effective method to treat HCC using small molecule inhibitor of NF-*κ*B. But this needs more evidence.

## 5. Conclusions

The main findings of our study are that PHF5A expression is significantly elevated in HCC tissues and cell lines, and the downregulation of PHF5A can inhibit the potential for migration and invasion in HCC cells. Further study demonstrated that PHF5A is implicated in NF-*κ*B signaling, and the transactivity of NF-*κ*B signaling is significantly inhibited in PHF5A-silenced HCC cells. The above results provide the evidence that PHF5A plays an important role in HCC progression and may be a novel therapeutic target of HCC.

However, there is a limitation in our study. For example, more cell lines and in vivo experiments are needed to study the role of PHF5A in HCC metastasis. We will investigate that in our future study.

## Data Availability

The data used to support the findings of this study are included within the article.

## References

[1] Bray F., Ferlay J., Soerjomataram I. (2018). Global cancer statistics 2018: GLOBOCAN estimates of incidence and mortality worldwide for 36 cancers in 185 countries. *CA: A Cancer Journal for Clinicians*.

[2] Torre L. A., Bray F., Siegel R. L., Ferlay J., Lortet-Tieulent J. (2015). Global cancer statistics, 2012. *CA: A Cancer Journal for Clinicians*.

[3] Wang H., Chen L. (2013). Tumor microenviroment and hepatocellular carcinoma metastasis. *Journal of Gastroenterology and Hepatology*.

[4] Ringelhan M., Pfister D., O'Connor T., Pikarsky E., Heikenwalder M. (2018). The immunology of hepatocellular carcinoma review-article. *Nature Immunology*.

[5] Llovet J. M., Zucman-Rossi J., Pikarsky E. (2016). Hepatocellular carcinoma. *Nature Reviews Disease Primers*.

[6] Hoesel B., Schmid J. A. (2013). The complexity of NF-*κ*B signaling in inflammation and cancer. *Molecular Cancer*.

[7] Didonato J. A., Mercurio F., Karin M. (2012). NF-*κ*B and the link between inflammation and cancer. *Immunological Reviews*.

[8] Perkins N. D. (2012). The diverse and complex roles of NF-*κ*B subunits in cancer. *Nature Reviews Cancer*.

[9] Sen R., Baltimore D. (1986). Multiple nuclear factors interact with the immunoglobulin enhancer sequences. *Cell*.

[10] Zhang Q., Lenardo M. J., Baltimore D. (2017). 30 Years of NF-*κ*B: A Blossoming of Relevance to Human Pathobiology. *Cell*.

[11] Karin M., Greten F. R. (2005). NF-*κ*B: linking inflammation and immunity to cancer development and progression. *Nature Reviews Immunology*.

[12] Taniguchi K., Karin M. (2018). NF-*κ*B, inflammation, immunity and cancer: coming of age. *Nature Reviews Immunology*.

[13] Hayden M. S., Ghosh S. (2014). Regulation of NF-*κ*B by TNF family cytokines. *Seminars in Immunology*.

[14] Shih R.-H., Wang C.-Y., Yang C.-M. (2015). NF-kappaB signaling pathways in neurological inflammation: A mini review. *Frontiers in Molecular Neuroscience*.

[15] Trappe R., Ahmed M., Gläser B. (2002). Identification and characterization of a novel murine multigene family containing a PHD-finger-like motif. *Biochemical and Biophysical Research Communications*.

[16] Trappe R., Schulze E., Rzymski T., Fröde S., Engel W. (2002). The Caenorhabditis elegans ortholog of human PHF5a shows a muscle-specific expression domain and is essential for C. elegans morphogenetic development. *Biochemical and Biophysical Research Communications*.

[17] Strikoudis A., Lazaris C., Trimarchi T. (2016). Regulation of transcriptional elongation in pluripotency and cell differentiation by the PHD-finger protein Phf5a. *Nature Cell Biology*.

[18] Teng T., Tsai J. H., Puyang X. (2017). Splicing modulators act at the branch point adenosine binding pocket defined by the PHF5A–SF3b complex. *Nature Communications*.

[19] Yang Y., Zhu J., Zhang T. (2018). PHD-finger domain protein 5A functions as a novel oncoprotein in lung adenocarcinoma. *Journal of Experimental & Clinical Cancer Research*.

[20] Zheng Y., Xue M., Shen H. (2018). PHF5A Epigenetically Inhibits Apoptosis to Promote Breast Cancer Progression. *Cancer Research*.

[21] Yang H., Cho M. E., Li T. W. H. (2013). MicroRNAs regulate methionine adenosyltransferase 1A expression in hepatocellular carcinoma. *The Journal of Clinical Investigation*.

[22] Nimmakayala R. K., Seshacharyulu P., Lakshmanan I. (2018). Cigarette Smoke Induces Stem Cell Features of Pancreatic Cancer Cells via PAF1. *Gastroenterology*.

[23] Hubert C. G., Bradley R. K., Ding Y. (2013). Genome-wide RNAi screens in human brain tumor isolates reveal a novel viability requirement for PHF5A. *Genes & development*.

[24] Guttridge D. C., Albanese C., Reuther J. Y., Pestell R. G., Baldwin A. S. (1999). NF-*κ*B controls cell growth and differentiation through transcriptional regulation of cyclin D1. *Molecular and Cellular Biology*.

[25] Rosa F. A. L. A., Pierce J. W., Sonenshein G. E. (1994). Differential regulation of the c-myc oncogene promoter by the NF-*κ*B Rel family of transcription factors. *Molecular and Cellular Biology*.

[26] Perkins N. D. (1997). Achieving transcriptional specificity with NF-kappa B. *The International Journal of Biochemistry & Cell Biology*.

[27] He Z., Dong W., Hu J., Ren X. (2017). AQP5 promotes hepatocellular carcinoma metastasis via NF-*κ*B-regulated epithelial-mesenchymal transition. *Biochemical and Biophysical Research Communications*.

[28] Tey S. K., Tse E. Y., Mao X. (2017). Nuclear Met promotes hepatocellular carcinoma tumorigenesis and metastasis by upregulation of TAK1 and activation of NF-*κ*B pathway. *Cancer Letters*.

